# Virtual Screening Technique Used to Estimate the Mechanism of *Adhatoda vasica* Nees for the Treatment of Rheumatoid Arthritis Based on Network Pharmacology and Molecular Docking

**DOI:** 10.1155/2020/5872980

**Published:** 2020-09-29

**Authors:** Wenxiang Wang, Yunsen Zhang, Jie Luo, Rushan Wang, Ce Tang, Yi Zhang

**Affiliations:** ^1^College Pharmacy of Chengdu University of Traditional Chinese Medicine, Chengdu 611137, China; ^2^Ethnic Medicine Academic Heritage Innovation Research Center of Chengdu University of Traditional Chinese Medicine, Chengdu 611137, China; ^3^Innovative Institute of Chinese Medicine and Pharmacy of Chengdu University of Traditional Chinese Medicine, Chengdu 611137, China

## Abstract

*Adhatoda vasica* Nees (AVN) is commonly used to treat joint diseases such as rheumatoid arthritis (RA) in ethnic minority areas of China, especially in Tibetan and Dai areas, and its molecular mechanisms on RA still remain unclear. Network pharmacology, a novel strategy, utilizes bioinformatics to predict and evaluate drug targets and interactions in disease. Here, network pharmacology was used to investigate the mechanism by which AVN acts in RA. The chemical compositions and functional targets of AVN were retrieved using the systematic pharmacological analysis platform PharmMapper. The targets of RA were queried through the DrugBank database. The protein-protein interaction network (PPI), Gene Ontology (GO), and Kyoto Encyclopedia of Genes and Genomes (KEGG) pathway enrichment analyses of key targets were constructed in the STRING database, and the network visualization analysis was performed in Cytoscape. Maestro 11.1, a type of professional software, was used for verifying prediction and analysis based on network pharmacology. By comparing the predicted target information with the targets of RA-related drugs, 25 potential targets may be related to the treatment of RA, among which MAPK1, TNF, DHODH, IL2, PTGS2, and JAK2 may be the main potential targets for the treatment of RA. Finally, the chemical components and potential target proteins were scored by molecular docking, and compared with the ligands of the protein, the prediction results of network pharmacology were preliminarily verified. The active ingredients and mechanism of AVN against RA were firstly investigated using network pharmacology. Additionally, this research provided a solid foundation for further experimental studies.

## 1. Introduction

Rheumatoid arthritis (RA), one of the major diseases leading to disability globally [1], is characterized by joint destruction, pannus formation, synovitis, and adjacent bone erosion [2]. RA can cause cartilage, bone damage, and even disability. RA, a chronic autoimmune disease, is characterized by joint inflammation, and is more like a syndrome that is composed of extra-articular manifestations, such as pulmonary involvement or vasculitis, rheumatoid nodules, and systemic comorbidities [3]. RA affects approximately 1% of the population, can present at any age, and bring enormous economic and social burden for both the individual and society [4]. Inflammation is the main factor to cause clinical symptoms in RA patients, so anti-inflammation is a key therapeutic strategy [5]. Many drugs such as nonsteroidal compounds, antirheumatic drugs, and glucocorticoids are used to treat RA [6–10]. However, these drugs are associated with many adverse effects and can only slow the progression of RA [11]. With developments in the field of medicine, the treatment of RA has entered a stage of diversified comprehensive treatment. Traditional Chinese medicine (TCM) as a supplemental and alternative drug might play a key role in treating RA [12].

*Adhatoda vasica* Nees (AVN), a popular Ayurvedic medicinal plant in India, is extensively distributed throughout India and tropical regions of South-East Asia including Tibet of China. AVN as an official drug listed in the Indian Pharmacopoeia is commonly used in the preparation of indigenous medicine to treat a variety of diseases such as asthma, cough, bronchitis, and tuberculosis [13]. Moreover, AVN is also commonly used in the treatment of RA in ethnic minority areas of China, especially in Tibetan and Dai areas.

Due to the multichemical components, multipharmacological effects, and multiaction targets of TCM in the treatment of diseases, the traditional research methods are difficult to completely uncover the mechanism of action [14]. However, the network pharmacology produced by the integration of bioinformatics and pharmacology in recent years can distinctly make clear the principle of action of such drugs and can systematically explain the role of multicomponent drugs in the treatment of diseases [15, 16]. Therefore, we will use the method of network pharmacology to study the effect of AVN in this paper; we used the method of network pharmacology to predict the target of chemical components of AVN, analyzed the interaction between target and metabolic pathway-related RA, and constructed the “component-target-metabolic pathway” network of RA, so as to provide reference for the further study of the material basis and mechanism of anti-RA. It not only provides some information support for the follow-up experimental research but also provides a new way and method for the research of Tibetan medicine.

**Figure 1:**
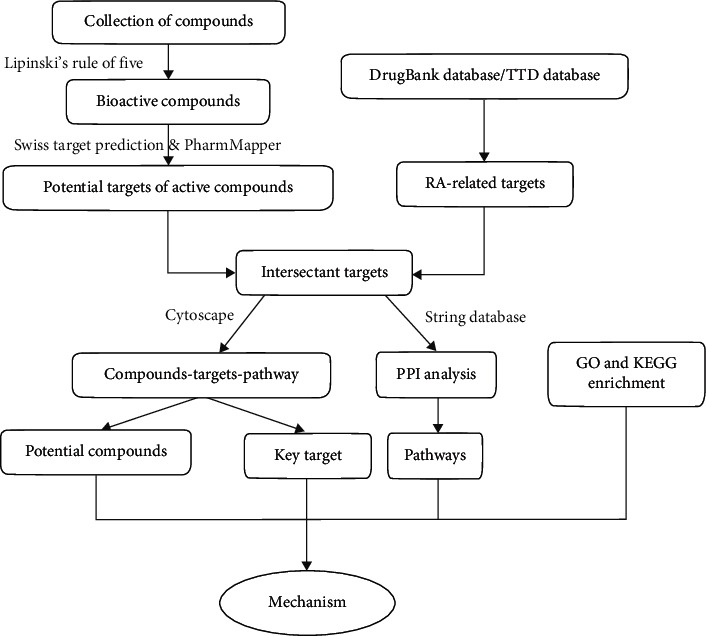
A flow chart of this study.

The workflow of this research on AVN against RA based on network pharmacology is shown in Figure 1.

## 2. Materials and Methods

### 2.1. Study Design

Chemical constituents of *Adhatoda vasica* Nees were collected from the public database and published research papers. Then, these compounds were preliminarily screened according to “Lipinski's rule of five.” Publicly available databases were utilized for determining human gene/proteins. Genes related to RA were solely selected, which were identified by human disease databases. Pathway analyses related to RA were performed again through publicly available databases. Finally, molecular docking was used to evaluate the interaction of active compounds acting on the targets.

### 2.2. Chemical Ingredients Database Building

A total of 53 compounds in AVN, including 27 alkaloids, 24 flavonoids, and 2 triterpenoids, were obtained from the previous studies in the public database and published research papers. SMILES formats for the compounds were obtained in the PubChem database [17]. For compounds that were not found in the PubChem database, SMILES formats were generated by ChemDraw (http://www.perkinelmer.com/category/chemdraw). Their PubChem ID and 2D chemical structures could be obtained on PubChem. Their CAS number could be obtained on the SciFinder database.

### 2.3. Active Compounds Screening

Traditional medical formulas have hundreds of compounds, but only a minority of them can produce a therapeutic effect. To identify potentially active compounds, we analyze the physicochemical properties of the compounds in the medicinal materials of AVN in this paper. Lipinski's rule of five is a rule of thumb to evaluate druglikeness or determine if a chemical compound with a certain pharmacological or biological activity has chemical properties and physical properties that would make it a likely orally active drug in humans. From the five criteria, molecular weight (MW), hydrogen bond acceptors (Hacc), hydrogen bond donors (Hdon), and octanol-water partition coefficient log P (AlogP) were considered, and hence, we have molecular weight (MW ≤ 500), Moriguchi octanol-water partition coeff (Log *P* ≤ 5), the number of donor atoms of H-bonds (HD ≤ 5), and the number of acceptor atoms for H-bonds (HA ≤ 10) [18].

### 2.4. Target Prediction

At present, whether active compounds could interact with targets is a critical stage to the drug discovery [19]. Accurate identification and validation of drug-target interactions is the first step on drug discovery pipeline [20]. By PubChem Compound (https://www.ncbi.nlm.nih.gov/pccompound/), we transformed the structure of the candidate compounds into SDF and Canonical SMILES structure format. Swiss Target Prediction (http://www.swisstargetprediction.ch/) [21] and PharmMapper server databases (http://lilab.ecust.edu.cn/pharmmapper) [22] with the “*Homo sapiens*” species setting were used for identification of the target genes linked to the selected constituents. The UniProt database (http://www) was utilized for retrieving gene information including name, gene ID, and organism. TTD (http://bidd.nus.edu.sg/BIDD-Databases/TTD/TTD.asp) [23] and DrugBank databases (https://www.drugbank.ca/) were searched for information on RA target genes using only “*Homo sapiens*” proteins linked to RA. Based on the above methods, 25 distinct targets associated with active constituents and RA were collected.

### 2.5. Network Construction

#### 2.5.1. GO and KEGG Enrichment Analysis of Targets Related to RA

To annotate the function of candidate genes and proteins associated with RA, the related biological processes, cellular components, molecular functions, and pathways were analyzed by online STRING 11.0 (https://string-db.org/cgi/help.pl?UserId=PWTj1MTAhQKH and session Id = 8wlBKy7kNz5I).

#### 2.5.2. Construction of PPI Network for RA Protein Targets

To construct the protein-protein interaction (PPI) network, online STRING 11.0 (https://string-db.org/cgi/help.pl?UserId=PWTj1MTAhQKH&sessionId=8wlBKy7kNz5I>) was used to analyze RA-related target proteins [24, 25]. Protein-protein interactions are critically important to many processes that take place in the cell, including regulation of gene expression, signal transduction, and cell migration. Afterwards, those RA-related targets were imported into STRING (version 11.0, https://string-db.org/) to investigate protein-protein interactions, and the targets with the species limited to “*Homo sapiens*” and interaction scores greater than or equal to 0.7 were used as the final targets to conduct GO and KEGG enrichment analysis and network construction.

#### 2.5.3. Construction of Compound-Target-Pathway Network

The network of compound-target-pathway was constructed using Cytoscape 3.7.0, an open software platform for network construction, analysis, and visualization, to identify the relationships of target proteins with each compound, the involved pathways, and diseases [26].

### 2.6. Molecular Docking

The crystal structure of screened targets was obtained from the RCSB PDB database. Maestro 11.1, a type of professional software, was used for verifying prediction and analysis based on network pharmacology and then conducting docking simulation and molecular pathway map. The candidate compounds were downloaded from PubChem and the SDF format using ChemDraw software was generated; candidate target proteins were transformed into PDB ID, and then, they were uploaded to Maestro 11.1 to get docking scores. A conventional rating of docking score ≥ 8.00 is considered very effective. However, in this paper, we innovatively proposed to use the ligand of protein as contrast, which greatly improved the accuracy of molecular docking results.

## 3. Results

### 3.1. Chemical Distribution of AVN

The effective information of selected compounds is demonstrated in Table S1. According to Lipinski's rule, those compounds whose MW was not more than 500 Daltons, AlogP and Hdon were not more than 5, and Hacc was not more than 10 were thought more likely to be the candidate drugs. However, some compounds, which might not meet this requirement but had significantly pharmacological activities supported by the literature, should also be adopted, such as kaempferol-3-O-rutinoside. The screening process of candidate compounds is shown in Table S2, and at length, 53 ingredients from AVN remained as 42.

### 3.2. Target Proteins of AVN

Searching for protein targets of AVN using traditional methods required a lot of manpower, material, and financial resources, and thus, the *in silico* model was utilized to provide a fast, efficient, and high-throughput approach to acquire the potential protein targets. Based on the pharmacophore matching method, some statistic factors, similarity measures, and so on, 344 targets of *H. sapiens* were obtained, in which PharmMapper contained 142 and Swiss contained 202, and then, the function of each target was obtained from UniProt and the published literature studies (Table S3).

### 3.3. Identification of RA Target Genes

The results of DrugBank and TTD databases retrieval illustrated that 199 genes were related to RA, and the detailed information is listed in Table S4.

### 3.4. GO and KEGG Enrichment Analysis for Targets

**Figure 2:**
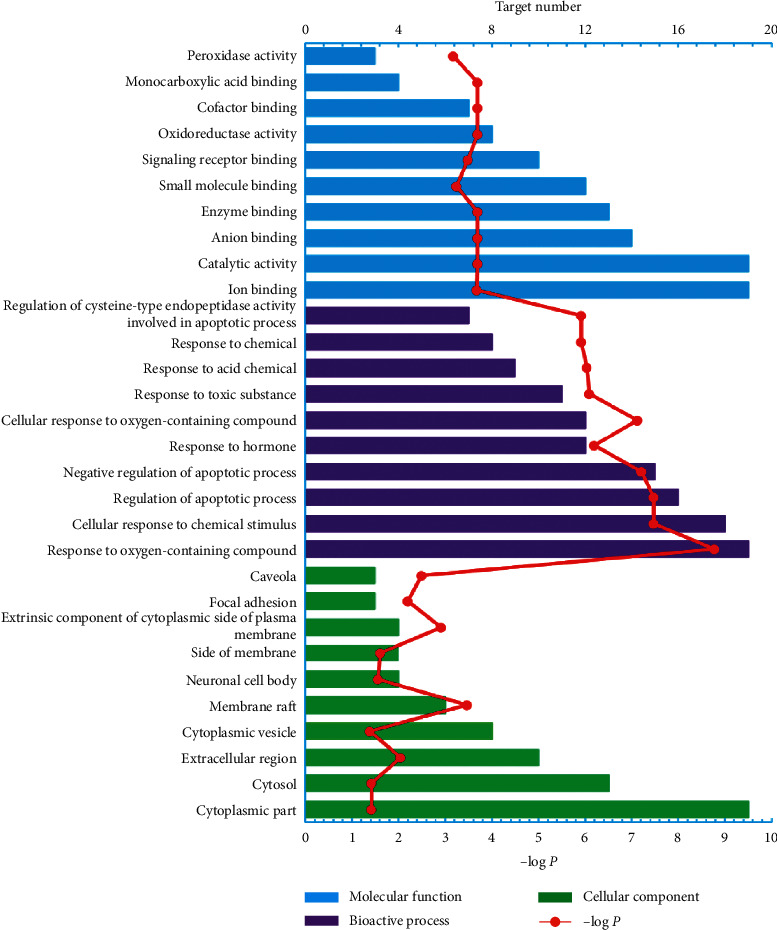
GO enrichment analysis of biological processes, cell composition, and molecular function annotation of the selected 25 target proteins.

Online STRING 11.0 was used to elucidate the biological processes, cell component, and molecular function annotation of the selected 25 targets (Figure 2). There are 611 GO entries (Figure 2 shows the top 10 according to FDR < 0.03), of which 547 entries are related to biological processes, including response to oxygen-containing compounds, regulation of apoptotic process, cellular response to chemical stimulus, negative regulation of apoptotic process, cellular response to oxygen-containing compounds, response to hormones, and response to toxic substances. 52 items are related to molecular functions, including catalytic activity, oxidoreductase activity, enzyme binding, monocarboxylic acid binding, anion binding, cofactor binding, ion binding, signaling receptor binding, and small molecule binding, and 12 cell component entries include membrane raft, focal adhesion, caveola, extrinsic components of the cytoplasmic side of plasma membrane, extracellular regions, side of membrane, neuronal cell body, cytosol, and cytoplasmic part (Table S5). Although a great deal of references manifest that the biological pathways involved in the target proteins of the chemical constituents in AVN are tightly bound to RA, further experimental verification still remains to determine the relationship between active compounds in AVN and their biological pathways.

**Figure 3:**
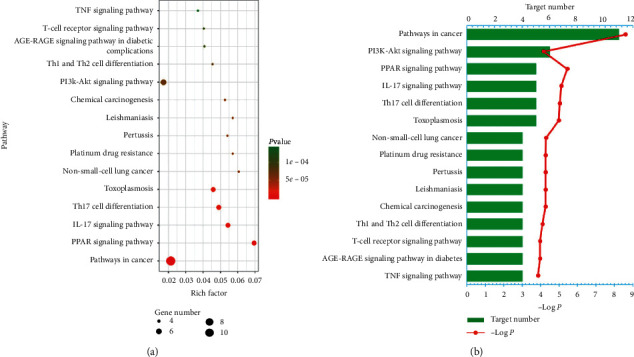
KEGG analysis of potential targets related to occurrence and development of rheumatoid arthritis: (a) Bubble chart and (b) Histogram.

To further determine the relationship between target proteins and biological pathways, we established a target-pathway network utilizing the online STRING database. 64 pathways corresponding to 25 protein targets were screened (Figure 3 shows the top 15 according to FDR < 0.0001) based on the KEGG analysis with FDR < 0.01, including pathways in cancer, PPAR signaling pathway, Th17 cell differentiation, toxoplasmosis, PI3K-Akt signaling pathway, and IL-17 signaling pathway. There is one target protein present in many pathways at a time, and several target proteins that exist in one pathway (Table S6).

Generally, one pathway involving many target proteins is more significant than one protein target interacting with many pathways. Hence, we should attempt to seek those pathways related to more target proteins. These results uncover that the active compounds of AVN may achieve the purpose of treating RA by regulating these signaling pathways.

### 3.5. Protein-Protein Interaction Network Construction

**Figure 4:**
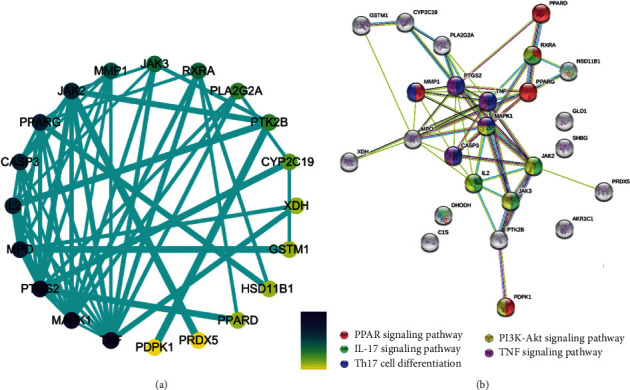
Protein-protein interaction network. (a) Constructed by Cytoscape 3.7.0; the color of the node is positively related to the degree of contribution of the node in the network. (b) Constructed by String 11.0; the color of the node is representative to the different signaling pathway as is shown above.

A PPI network (Figure 4) was constructed in order to clarify the relationship between the 25 RA-related targets (Table 1). The nodes in the disease PPI network represent the interrelationships during the development of RA. We constructed a total of 20 nodes and 56 edges in the PPI network of RA. The color of the node is positively related to the degree of contribution of the node in the network. The color of nodes such as MAPK1, TNF, IL2, PTGS2, and JAK2 are darker and can be easily found in the PPI network, and the corresponding degree values (Table 2) of each node are 17, 14, 12, 12, and 10. These targets may play a key role in the development of RA disease.

From the above results, it is obvious that the five proteins of MAPK1, TNF, IL2, PTGS2, and JAK2 are located at the crucial position, which are mainly involved in MAPK, TNF, JAK, and PI3K-Akt signaling pathways, thus regulating the occurrence and development of RA. Among them, TNF-*α* has a pleiotropic effect on cell growth, differentiation, cell killing, and inflammation. It also involves the pathogenesis of various autoimmune diseases, such as RA [27], which plays an important role in islet *β*-cell destruction. More reported evidence displayed that TNF-*α* can induce apoptosis in certain cell types [28], whereas TNF-*α*-activated MAPK is required for TNF-*α*-induced apoptosis.

### 3.6. Compound-Target-Pathway Network Analysis of AVN

**Figure 5:**
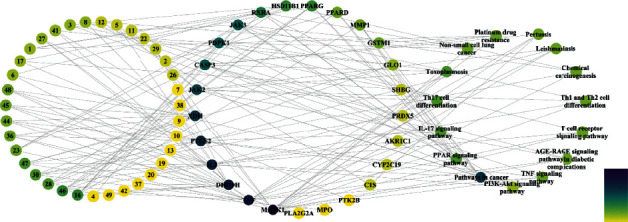
Component-target-pathway network (The color of the node is positively related to the degree of contribution of the node.).

To further clarify the potential targets, a component-target-pathway network was established based on the network pharmacology as shown in Figure 5. The network embodies chemical components, target proteins, and metabolic pathways, consisting of 75 nodes and 163 edges. 35 components interact with 25 target proteins and are associated with RA through 15 pathways. Compared with all the target proteins mentioned above, the 25 target proteins in this network are more possibly to be the potential targets of AVN. However, a great quantity of animal experiments and clinical trials are still needed to verify these predictions.

### 3.7. Molecular Docking

**Figure 6:**
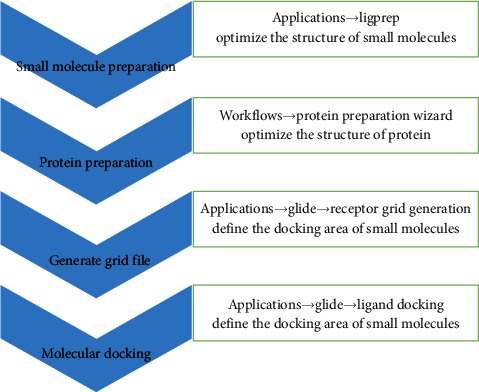
Procedure of molecular docking.

**Figure 7:**
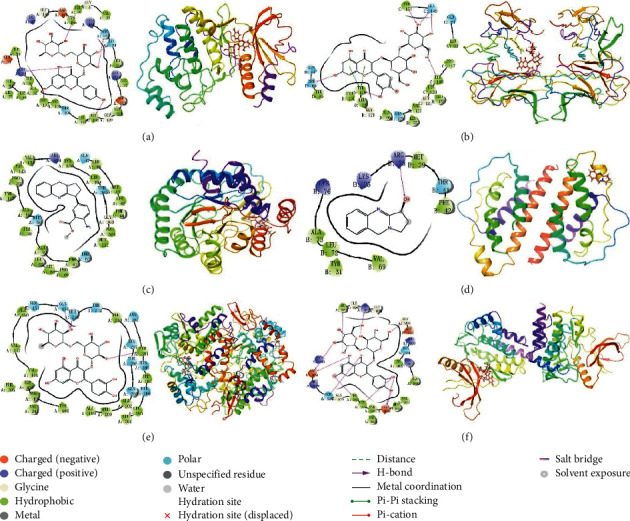
Molecular docking of active compounds and key targets: (a) kaempferol-3-O-rutinoside to MAPK1; (b) quercetin-3-O-sophoroside to TNF; (c) N-demethyl adhatodine to DHODH; (d) vasicine to IL2; (e) quercetin-3-O-rutinoside to PTGS2; and (f) quercetin-3-O-sophoroside to JAK2.

A total of 42 compounds were selected for docking on the 6 target proteins under the procedure as shown in Figure 6, and the molecular docking scores of the compounds are summarized in Table S7. The score of the small molecular ligand docking on the target protein complex was referred to as the threshold, so these compounds docking on this protein with a score greater than this threshold value are considered as the active compounds acting on the target. Finally, we summarize the top three chemical components bound to the key target displayed in Table 3, and the highest scoring compound with key proteins is visualized by molecular docking in Figure 7.

## 4. Discussion

The network pharmacology technique is relatively novel and was proposed by Li et al. for the first time [29]. It provides a deeper understanding of systems biology and network theory, and it has been considered the extra paradigm in drug discovery [30–34]. Network pharmacology has been used to investigate metabolic pathways between drugs and target proteins or genes and diseases, and it can describe complexities among biological systems, drugs, and diseases from informatics perspective [35–37]. Therefore, the development of network pharmacology interacting with molecular docking techniques that can predict multiple drug–target relationship may achieve future drug discoveries in complex diseases such as RA. Here, we integrated various information from publicly available databases to predict the mechanism between AVN and its potential targets related to RA, as well as the signaling pathways involved. Increased mortality in RA is widely recognized [38], so it is very significant to develop safe and effective drugs to antagonize RA. TCM has an irreplaceable effect in treatment of RA in China [39]. However, the active compounds and mechanisms of TCM against RA remain still unclear, and AVN is one of them. In this paper, network pharmacology and molecular docking were employed to tackle these issues, and the results of the present study illuminated the active compounds and mechanisms of AVN against RA based on the holistic perspective and the characteristic of TCM.

The KEGG official website indicated that inflammatory cell infiltration, synovial pannus formation, angiogenesis, bone resorption, and joint destruction are the crucial biological effects involved in RA signaling pathways, and these biological effects were directly related to RA pathogenesis [40–42].

Previous investigations indicated that synovial inflammatory cells were obviously decreased after the anti-TNF-*α* mAb treatment, manifesting that TNF-*α* played an important role in RA pathogenesis [43–45]. The pathogenesis of RA was overexpressed inflammatory cytokines and tissue injury mediated by NF-*κ*B activation, and drugs could alleviate symptoms of RA by blockade of NF-*κ*B activation [46, 47]. Recent work demonstrated that the insufficient apoptosis of inflammatory cells in an RA patient might contribute to pathogenesis and induction of inflammatory cell apoptosis is a feasible strategy for treating RA [48]. These reports confirmed the correctness and rationality of prediction of the molecular mechanisms of AVN against the RA network based on pharmacology. The relationships between other signaling pathways and RA were not discussed in detail in this work, but their relationships could be easily identified by retrieving literature data.

The degree value of genes indicated their contribution to the therapeutic effect of AVN on RA, and that PTGS2 was the core gene of AVN against RA. In the 64 signaling pathways, PTGS2 was enriched in TNF and NF-*κ*B signaling pathways, and the KEGG official website uncovers the effect of PTGS2 in the two signaling pathways by regulating inflammation response. COX-2 enzyme, generated by PTGS2 gene, was induced by proinflammatory cytokines to promote inflammation progression, and its expression was selectively blocked by dexamethasone, a potent anti-inflammatory drug [49]. Inflammation is the crucial factor to trigger RA symptoms, such as joint damage, disability, and comorbidity; hence anti-inflammation is the main therapeutic strategy [5]. The key targets in the compound-target network of AVN are PTGS2 and MAPK1. These targets are involved in various aspects of disease regulation such as inflammation, control, and endocrine therapy of tumors. PTGS is a key rate-limiting enzyme involved in the synthesis of prostaglandins from arachidonic acid. There are two isomerases: PTGS1 and PTGS2. PTGS2 plays an important role in the development of RA [50]. Studies have shown that the expression of cyclooxygenase is significantly increased in the synovial tissue of patients, accompanied by the induction of prostaglandin E2, and inflammatory factors are synthesized in large quantities. This causes the infiltration of inflammatory cells, abnormal proliferation of synovial tissue, and formation of neovascularization, resulting in swelling and degeneration of joints [51]. Therefore, by inhibiting the expression of PTGS2 in synovial cells, the synthesis of prostaglandin E2 can be reduced, consequently reducing the inflammatory response of RA and improving the disease [52]. Therefore, AVN or the combination of the above components for some key targets plays an important role in the regulation of inflammatory response. TNF-*α* can effectively reduce the arthritis and synovitis symptoms of RA patients, and RA can be suppressed by inhibiting the expression of TNF-*α* [43].

It was reported that andrographolide demonstrated protective effects on RA through regulating MAPK pathways, suggesting that the MAPK signaling pathway was related to occurrence and development of RA [53]. The PI3K-Akt signaling pathway might be the hub signaling pathway of AVN against RA. Joint synovium is the main diseased region in RA patients. Therefore, inducing apoptosis of synovial cells is a feasible strategy for treating RA by preventing development of inflammation [54]. The PI3K-Akt signaling pathway was abnormally activated in RA synovium, resulting in the overexpression of antiapoptotic genes such as FLIP, Bcl-2, and Mcl-1 [55]. The overexpression of these antiapoptotic genes results in out-of-balance apoptosis of synovial cells, which induced RA [56]. Additionally, luteolin, an uppermost active ingredient against RA, inhibited the proliferation of synovial fibroblasts in RA by blocking the PI3K-Akt signaling pathway [57]. PPARs (peroxisome proliferation-activated receptors) are ligand-activated transcription factors. PPAR-*γ*, a subtype of PPARs, is more closely related to RA. The expression of PPAR-*γ* can be detected in synovial cells involved in RA. PPAR-*γ* agonists can inhibit the hyperplasia of synovial cells and induce their apoptosis [58, 59]. In addition, PPAR-*γ* agonists can inhibit the generation of key mediators in RA from macrophages, including IL-1*β*, IL-6, and TNF-*α*. In conclusion, the PPAR signaling pathway is important in treating RA by intervening with the pathological process of RA through the corresponding receptor agonists. Based on these existing reports, it is reasonable to conclude that the mechanism of AVN against RA was inflammation response through inactivating TNF, MAPK, PPAR, and PI3K-Akt signaling pathways.

The pathophysiological mechanism of RA is specially complicated, and various biological processes and metabolic pathways are involved in the process of RA damage [60]. 198 RA targets screened in this study mainly partake in the release of inflammatory cytokines and proinflammatory factors by TNF, MAPK, and PI3K-AKT signaling pathways [61, 62]. According to the network of “active compound-target-pathway,” AVN may intervene the inflammation pathways to reduce the release of proinflammatory factors and inflammatory cytokines through “multicomponent-single target” or “multicomponent-multitarget”. Collectively, AVN may have significant potential to treat RA by a combination of multicomponents, multitargets, and multipathways.

As TCM is characterized by multicomponent, multitarget, multilevel, and network-based therapeutic effects, traditional pharmacy, pharmacology, and molecular biology research are not enough to explain its complex mechanism and material basis. Based on the big data platform of network pharmacology research (a database involving multiple drug components, disease targets, protein interactions, and signal pathways), the overall analysis of the material basis and mechanism of action is not only comprehensive but also conducive for refining its main therapeutic efficacy-related targets, components, pathways, and a comprehensive view of the problem. However, there were some limitations to this study. For example, although there are some clinical studies on the anti-inflammatory and immunomodulatory effects of the compounds analyzed, these mechanisms need to be further verified. Although network pharmacology is a simple and efficient method for predicting drug targets in sophisticated diseases, it is still necessary to verify the scientific nature and rationality of predicted targets by in vitro experiments.

## 5. Conclusion

This paper firstly explored the active compounds and molecular mechanisms of AVN against RA based on network pharmacology and molecular docking. The active chemical constituents of AVN against RA consisted of 35 compounds, and peganidine, quercetin-3-*O*-rutinoside, and quercetin were the key active ingredients. The related genes of AVN against RA included 25 target genes, and MAPK1 and PTGS2 were the hub genes. The mechanism of AVN against RA mainly comprised 15 signaling pathways, and the key mechanism was related to inhibition of inflammation response through inactivating TNF and PPAR signaling pathways. Additionally, this research provided scientific evidence and a good theoretical foundation to support the therapeutic effect of AVN on RA. Although we predicted the possible target proteins by AVN and further verified them by molecular docking, the two methods are prediction after all. Then, we prepare to use effective fraction or active compounds to treat synovial cells in human arthritis, analyzing expression levels on proteins and mRNA levels by transcriptomics and proteomics studies. At length, our results predict that the therapeutic effects of AVN against RA are mediated via MAPK1, TNF, IL2, PTGS2, DHODH, and JAK2. These results may be useful in guiding further research to clearly clarify the molecular targets of AVN related to RA and applications of network pharmacology in drug discovery.

## Figures and Tables

**Table 1:** RA-related potential target information.

Number	UniProt name	Target	Gene	Frequency
1	Q02127	Dihydroorotate dehydrogenase (quinone), mitochondrial	DHODH	13
2	P47989	Xanthine dehydrogenase/oxidase	XDH	11
3	P35354	Prostaglandin G/H synthase 2	PTGS2	7
4	P01375	Tumor necrosis factor	TNF	7
5	P60568	Interleukin-2	IL2	7
6	P28845	Corticosteroid 11-beta-dehydrogenase isozyme 1	HSD11B1	5
7	P28482	Mitogen-activated protein kinase 1	MAPK1	5
8	O15530	3-Phosphoinositide-dependent protein kinase 1	PDPK1	4
9	P37231	Peroxisome proliferator-activated receptor gamma	PPARG	4
10	P52333	Tyrosine-protein kinase JAK3	JAK3	4
11	Q04760	Lactoylglutathione lyase	GLO1	3
12	P19793	Retinoic acid receptor RXR-alpha	RXRA	3
13	O60674	Tyrosine-protein kinase JAK2	JAK2	3
14	P05164	Myeloperoxidase	MPO	2
15	Q04828	Aldo-keto reductase family 1 member C1	AKR1C1	2
16	P30044	Peroxiredoxin-5, mitochondrial	PRDX5	2
17	P04278	Sex hormone-binding globulin	SHBG	2
18	Q03181	Peroxisome proliferator-activated receptor delta	PPARD	2
19	P42574	Caspase-3	CASP3	2
20	P33261	Cytochrome P450 2C19	CYP2C19	1
21	Q14289	Protein-tyrosine kinase 2-beta	PTK2B	1
22	P03956	Interstitial collagenase	MMP1	1
23	P09488	Glutathione S-transferase Mu 1	GSTM1	1
24	P14555	Phospholipase A2, membrane associated	PLA2G2A	1
25	P09871	Complement C1s subcomponent	C1S	1

**Table 2:** The degree value of the target proteins.

Target	Abbreviation	UniProt ID	PDB ID	Degree
Tyrosine-protein kinase JAK2	JAK2	O60674	4Z32	10
Dihydroorotate dehydrogenase (quinone), mitochondrial	DHODH	Q02127	5TCE	13
Mitogen-activated protein kinase 1	MAPK1	P28482	4QP3	17
Interleukin-2	IL2	P60568	3QB1	12
Tumor necrosis factor	TNF	P01375	5MU8	14
Prostaglandin G/H synthase 2	PTGS2 (COX2)	P35354	5F1A	12

**Table 3:** The docking score of the top three chemical components bound to the key targets.

Protein	Chemical constituents	Glide gscore	Glide hbond	Glide evdw	Glide ecoul	Glide energy
MAPK1	Contrast	−8.969	−0.32	−45.135	−5.983	−51.118
	Kaempferol-3-O-rutinoside	−9.947	−0.551	−56.092	−25.812	−81.904
	Quercetin-3-O-rutinoside	−9.385	−0.315	−63.010	−18.544	−81.554
	Kaempferol-3-O-glucoside	−8.865	−0.509	−46.273	−19.352	−65.624

TNF	Contrast	−7.971	0	−45.563	−1.936	−47.499
	Quercetin-3-O-sophoroside	−7.968	−0.32	−42.107	−19.831	−61.938
	Kaempferol-3-O-rutinoside	−7.905	−0.289	−43.940	−15.03	−58.969
	Deoxyaniflorine	−7.782	0	−37.262	−1.324	−38.586

DHODH	Contrast	−14.062	−54.687	−9.844	−64.531	−54.687
	N-Demethyl adhatodine	−10.131	0	−51.576	0.051	−51.525
	Adhatodine	−10.077	0	−48.886	0.204	−48.682
	Deoxyaniflorine	−10.01	−0.535	−19.431	−9.260	−28.691

IL2	Contrast	−6.76	−0.72	−35.565	−13.090	−48.655
	Vasicine	−5.971	0	−22.876	−1.874	−24.750
	Vasicine acetate	−5.89	−0.182	−23.229	−4.451	−27.680
	1,2,3,9-Tetrahydro-5-methoxypyrrolo-ol	−5.831	−0.037	−25.257	−2.302	−27.559

PTGS2	Contrast	−10.182	0	−41.023	−10.356	−51.379
	Quercetin-3-O-rutinoside	−10.301	0	−64.482	−11.75	−76.232
	Kaempferol-3-O-sophoroside	−9.721	−0.154	−52.359	−20.364	−72.723
	Kaempferol-3-O-rutinoside	−9.589	0	−60.938	−12.074	−73.013

JAK2	Contrast	−10.660	−0.569	−41.471	−7.018	−48.490
	Quercetin-3-O-sophoroside	−10.520	−0.174	−49.761	−23.978	−73.739
	Orientin	−9.740	−0.144	−31.871	−27.504	−59.376
	Quercetin	−9.441	−0.603	−32.308	−18.148	−50.457

Note: Glide hbond stands for contribution of hydrogen-bonding. Glide evdw stands for van der Waals energy. Glide ecoul stands for Coulomb energy. All items are involved in the Glide gscore algorithm.

## Data Availability

The data used to support the findings of this study are included within Supplementary Materials.

## Supplementary Materials

Supplementary Materials Table S1: effective information of selected compounds. Table S2: screening of candidate compounds. Table S3: a list of genes linked to all candidate compounds from *Adhatoda vasica* Nees. Table S4: a list of 199 genes linked to RA from DrugBank and TTD database. Table S5: the biological processes, cell composition, and molecular function annotation by String database of the selected 25 target proteins. Table S6: the KEGG analysis by String database of the selected 25 target proteins. Table S7: the docking score of all chemical constituents and MAPK1, IL2, TNF, PTGS2, DHODH, and JAK2.
